# Targeted Metabolomic Analysis Reveals Solvent-Dependent Phenolic Variation and Associated Antioxidant and Antibacterial Activity in Coriander Seeds (*Coriandrum sativum* L.)

**DOI:** 10.3390/molecules30224387

**Published:** 2025-11-13

**Authors:** Charitha P. Ekanayake, Joel Johnson, Ryan J. Batley, Ryan du Preez, Tieneke Trotter, Joseph Robert Nastasi, Padraig Strappe, Daniel Broszczak, Mani Naiker

**Affiliations:** 1 Jawun Research Institute, CQUniversity, Rockhampton, QLD 4701, Australia; charitha.priyadarshani@cqumail.com (C.P.E.); joel.johnson@uq.edu.au (J.J.); ryan.batley@cqumail.com (R.J.B.); r.dupreez@cqu.edu.au (R.d.P.); t.trotter@cqu.edu.au (T.T.); 2 Centre for Nutrition and Food Sciences, Queensland Alliance for Agriculture and Food Innovation (QAAFI), The University of Queensland, Brisbane, QLD 4108, Australia; 3 Institute of Future Farming, CQUniversity, Rockhampton, QLD 4701, Australia; 4 School of Agriculture and Food Sustainability, The University of Queensland, Brisbane, QLD 4072, Australia; j.nastasi@uq.edu.au; 5 School of Medicine, Charles Darwin University, Darwin, NT 0909, Australia; 6 School of Biomedical Sciences, Faculty of Health, Queensland University of Technology, Brisbane, QLD 4000, Australia; daniel.broszczak@qut.edu.au

**Keywords:** coriander (*Coriandrum sativum* L.) seeds, phenolics, antioxidant capacity, antibacterial activity, extracts

## Abstract

*Coriandrum sativum* L. (coriander) seeds have long been used as a traditional medicine and a spice worldwide. Despite its abundant use, bioactive properties of coriander seeds, correlated with the antioxidant and phenolic data, have not been fully studied systematically. This study evaluated the phenolic data, antioxidant capacity, and antibacterial activity of coriander seed extracts obtained using solvents of differing polarity. Higher total phenolic content (TPC) and antioxidant activity were observed in the polar extracts, while moderate and non-polar extracts possessed higher antibacterial activity. Acetone extract (Ace) had the highest antibacterial activity, with an inhibition zone diameter (IZD) of 16.2 ± 0.2 mm against *B. subtilis*, and a minimum inhibitory concentration (MIC) and minimum bactericidal concentration (MBC) of 1 and 2% (*w*/*v*), respectively. The MBC/MIC ratio between 1 and 4 was observed for the active extracts of coriander seeds, indicating their bactericidal behavior. The liquid chromatography and tandem mass spectroscopy (LC–MS/MS) system using multiple reaction monitoring (MRM) targeted analysis identified 13 phenolic compounds: gallic acid, ellagic acid, gentisic acid, caffeic acid, vanillic acid, o-coumaric acid, sinapinic acid, chlorogenic acid, salicylic acid, ferulic acid, rutin, trans-cinnamic acid, and quercetin. Quantitative differences were observed in the phenolic compounds across the different coriander seed extracts. The TPC was significantly (*p* ≤ 0.01) and positively correlated with cupric-reducing antioxidant capacity (CUPRAC) (r = 0.92), as well as with ferric-reducing antioxidant power (FRAP) (r = 0.98); furthermore, it showed that the higher level of antioxidant capacity of the coriander seed extracts was significantly (*p* ≤ 0.05) associated with phenolic compounds such as gallic acid, chlorogenic acid, gentisic acid, ferulic acid, and rutin. However, antibacterial activity and phenolic/antioxidant content were negatively correlated, suggesting that non-polar compounds may impact antibacterial activity.

## 1. Introduction

*Coriandrum sativum* L. (family Apiaceae) is a well-known annual herb, commonly known as coriander in English, native to the Mediterranean and Middle Eastern regions of the world. Cultivated primarily for its seeds, coriander has a rich history of use as a spice and flavoring agent [1]. Furthermore, coriander seeds have long been used in traditional medicine due to the presence of health-promoting compounds. They are considered effective in the treatment and management of many diseases such as influenza, gastrointestinal issues, urinary tract disorders, cardiovascular diseases, diabetes, and rheumatism [1,2,3].

These therapeutic effects are mainly attributed to bioactive secondary metabolites. Coriander seeds are a rich source of essential oils, lipids, and phenolic compounds, particularly hydroxybenzoic acids (protocatechuic acid, vanillic acid, and gallic acid), hydroxy cinnamic acids (chlorogenic acid, caffeic acid, and p-coumaric acid), and flavonoids (catechin/epicathechin, epicathechin gallate, gallocathechin/epigallocathechin, and rutin) [4]. Coriander-seed essential oil is a complex mixture of volatile compounds, including oxygenated monoterpenes (linalool, camphor, geraniol, and geranyl acetate), monoterpene hydrocarbons (α-pinene, camphene, p-cymene, limonene, and γ-terpinene), and sesquiterpenes (caryophyllene); among these, linalool is the most abundant (45.38–81.7%) volatile compound [5,6]. Such secondary metabolites of coriander seeds may facilitate radical scavenging when consumed, leading to a decrease in antioxidant stress, which is a prominent risk factor for various diseases.

Previous studies have reported that coriander seeds possess antioxidant, antidiabetic, anti-inflammatory, anti-atherosclerosis, anti-arrhythmic, anthelmintic, and antibacterial bioactive properties [7,8]. The antibacterial properties of coriander seeds have primarily been studied with non-polar extracts/fractions/compounds such as coriander-seed oil, essential oil, and its major constituents. The essential oil obtained from coriander seeds has demonstrated effectiveness against Gram-positive and -negative bacteria, including *Bacillus cereus*, *Micrococcus luteus*, *Staphylococcus aureus*, *Salmonella typhimurium*, and *Escherichia coli* [9]; equally, its major constituents (linalool, geranyl acetate, α-pinene, and p-cymene) have exhibited antibacterial activity against the antibiotic-resistant bacterium *Acinetobacter baumannii* [10], with linalool showing the best antibacterial activity. Seed oil of coriander seeds has also inhibited the growth of Gram-positive and -negative bacteria such as *Klebsiella pneumonia*, *Pseudomonas aeruginosa*, and methicillin-resistant *Staphylococcus aureus* (MRSA) [11]. However, studies on the antibacterial properties of polar extracts from coriander seeds have been limited [12,13]. Therefore, it is important to carry out a systematic and comparative evaluation of the antibacterial properties of both the polar and non-polar extracts of coriander seeds, which may lead to identifying its most potent antibacterial extract/s.

The extraction of bioactive compounds from plant materials using solvents of different polarity is important as it chemically fractionates compounds. For instance, plant extracts prepared from polar solvents such as water, methanol, and ethanol contain hydrophilic compounds such as phenolic acids and flavonoids, while those prepared with low-polarity solvents like hexane contain less lipophilic compounds, such as terpenes [14]. Therefore, plant extracts prepared with different solvents carry different bioactive compounds that can exhibit distinct antibacterial and other biological properties.

The objective of this study is to evaluate and compare the total phenolic content, antioxidant properties, phenolic profiles, and antibacterial activity of polar and non-polar coriander seed extracts against some common food-borne bacteria. It paves the way to identify the coriander seed extracts that show antioxidant and antibacterial activities, which have potential implications for the food and nutraceutical industries. Finally, by identifying and quantifying the most abundant phenolic compounds in coriander seed extracts obtained with solvents of differing polarity, optimal extraction methods that target-specific compounds may be elucidated.

## 2. Results and Discussion

### 2.1. Total Phenolic Content (TPC) and Antioxidant Capacity of Coriander Seeds

The phenolic content of two commercial coriander-seed samples was determined in terms of total phenolic content (TPC) (Table 1). According to the results, the TPC of two commercial coriander-seed samples, denoted “In” and “Hy”, were 274 ± 3 and 161 ± 3 mg GAE/100 g dry weight (DW), respectively. A study by Tibebe et al. [15] reported the TPC of 70% aqueous methanolic extract of coriander seeds (prepared by maceration), originating in Ethiopia, as low as 179.9 mg GAE/100 g DW, while the study by Hameed et al. [16] reported that the TPC of coriander seeds sourced from Pakistan was as high as 289.3 mg GAE/100 g DW following the same extraction solvent and method. Another study has reported that the TPC of pure methanol extracts (extracted by maceration) of three coriander-seed varieties—sourced from Tunisia, Syria, and Egypt—varies at 100, 109, and 94 mg GAE/100 g DW, respectively [17]. A study by Ianni et al. [18] investigated the TPC of coriander seed extracts (origin: Bulgaria) prepared from maceration and ultrasound-assisted extraction techniques (40 min) using a choline chloride-based natural deep eutectic solvents (NADES) system. According to the results, the TPC of the coriander seed extracts was 158.4 and 183.5 mg GAE/100 g DW, respectively [18]. Overall, the variation in phenolic content in coriander seeds may be attributed to various factors such as geographical distribution [15,16], different varieties/genotypes [17], processing/storage conditions [19], and extraction methods [18].

The antioxidant capacity of seed samples was determined by the cupric-reducing antioxidant capacity (CUPRAC) and ferric-reducing antioxidant power (FRAP) assays. The CUPRAC of “In” and “Hy” are 1142 ± 13 and 620 ± 20 mg TXE/100 g DW, respectively, while the FRAP of the two samples are 424 ± 13 and 279 ± 10 mg TXE/100 g DW, respectively (Table 1). A study by Demir & Korukluoglu [20] reported the CUPRAC and FRAP of commercial Turkish coriander seeds as 23 and 36.2 mM Trolox/kg DW (equivalent to 575.7 and 905 mg TEX/100 g DW, respectively). However, in our study, the coriander-seed samples reported higher CUPRAC antioxidant potential than FRAP. Dissimilarity between reported values can be attributed to the sample preparation method and extraction solvent as it affects the distribution of antioxidant compounds in the extract [21]. Moreover, the sample origin and processing conditions will also have an effect, as discussed earlier.

### 2.2. Extraction Yield of Different Extracts of Coriander Seeds

The “In” sample was selected to prepare extracts, and was further studied as it has higher phenolic and antioxidant capacity compared to the “Hy” sample. The yields of coriander seed extracts are reported in Table 2. Extraction yields varied greatly depending on the solvent, in decreasing order of Et (ethanol extract) > He (hexane extract) > EA (ethyl acetate extract) > W (water extract) > Ace (acetone extract) > M_1_ (methanol extract 1) > M_2_ (methanol extract 2) (Table 2). Previous work has found that the yield of methanol extract (100% methanol) of seeds from different coriander genotypes varies between 3.1 and 4.8% [22], which is lower than in the present study. The study by Balbino et al. [23] revealed that the percentage yield of oil (a non-polar fraction) from coriander seeds was 12.30% (*w*/*w*), which is aligned with the yield of the non-polar extract (He) in our study [12% (*w*/*w*)]. As the fats of coriander seeds are well-extracted with the hexane solvent, the yield of He is reasonably comparable with the fat content of the seeds. A study by Shahwar et al. [24] showed that 100 g of coriander seeds contains 12.58 g of protein, 9.12 g of fat, and 37.14 g of fiber. As such, the fat content of coriander seeds in the present study is comparable with the previous literature. The highest extraction yield was reported for Et, which is 12.1% (*w*/*w*). According to the previous literature, the extraction yield of coriander seeds using 100% ethanol by maceration is 2.17% (*w*/*w*). The difference in the extraction yields may be due to the aqueous content of ethanol, the extraction technique used, and the origin of the coriander seeds [25].

### 2.3. In Vitro Antibacterial Activity of Coriander Seed Extracts

#### 2.3.1. Inhibition Zone Diameter of Different Coriander Seed Extracts

**Figure 1:**
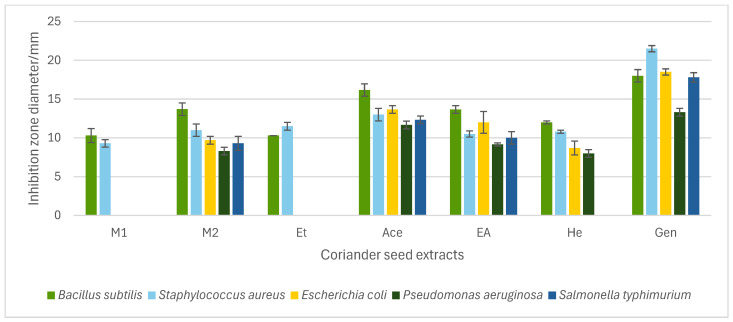
Antibacterial activity of coriander seed extracts [methanol (M_1_, M_2_), ethanol (Et), acetone (Ace), ethyl acetate (EA), and hexane (He)] and gentamicin (Gen) measured as inhibition zone diameter in the agar-well diffusion assay. The M_1_, M_2_, Et, Ace, EA, He extracts were tested at 30% (*w*/*v*). Data represent means of triplicate analysis ± SD. The graph only includes coriander seed extracts that exhibited inhibition against at least one bacterium.

The in vitro antibacterial potential of different coriander seed extracts (W, M_1_, M_2_, Et, Ace, EA, and He) was investigated against five food-borne bacteria. The coriander seed extracts exhibited varying degrees of antibacterial activity against tested bacteria, depending on the type of the extract. The growth inhibition/inhibition zone diameter can be used to screen the antibacterial activity of coriander seed extracts. Six out of seven tested extracts showed growth inhibition against at least one food-borne bacteria: M_1_, M_2_, Et, Ace, EA, and He (Figure 1). The W, which was prepared from cold water, did not show any growth inhibition against the bacteria tested. The inhibition zone diameter of extracts varied from 8.3 ± 0.5 mm (M_2_ against *Pseudomonas aeruginosa*) to 16.2 ± 0.2 mm (Ace against *Bacillus subtilis*). The Ace, EA, and M_2_ extracts were effective in inhibiting the growth of all tested Gram-positive and -negative bacteria, indicating that both polar and non-polar extracts of coriander seeds contain active compounds.

The previous literature has reported the antibacterial activity of polar extracts of coriander seeds. According to one study, a 50% ethanol extract of coriander seeds showed a positive effect against *B. cereus* (9.3 ± 0.3 mm) but no activity against *Staphylococcus aureus*, *Escherichia coli*, and *P. aeruginosa* [12]. Similarly, results from our study show that *B. subtilis* (10.3 ± 0.47 mm) is susceptible to Et (80% ethanol), while *E. coli* and *P. aeruginosa* are not sensitive to it. In addition, our study shows that Et inhibits the growth of *S. aureus* (11.5 ± 0.4 mm); similar to our results, the study by Susanti et al. [26] reported antibacterial activity of ethanol extract (96%) of coriander seeds against *S. aureus* (growth inhibition of 11.7 mm). Previous analysis by Zeynel et al. has reported the antibacterial potential of methanol (96%) extract of coriander seeds prepared from maceration against *E. coli*, *S. aureus*, *P. aeruginosa*, *Klebsiella pneumoniae*, and *Acinetobacter baumani* [27]. Our results show that M_1_ (90% methanol extract) was effective against *B. subtilis* and *S. aureus*, while M_2_ (70% methanol extract) was active against all bacteria tested. The difference in the growth inhibitions in methanol extracts of coriander seeds may be attributed to the composition of the extraction solvent and the various extraction methods employed. The water extract from coriander seeds (W) did not possess any antibacterial potential against all tested bacteria. Similar to our results, previous work has reported that water extract of coriander seeds does not show any inhibition against *E. coli*, *S. aureus*, and *P. aeruginosa* [13].

An antibacterial effect from the Ace, EA, and He extracts was observed against all bacteria tested, except He against *Salmonella typhimurium*. Prior work has indicated that acetone extract of coriander seeds has shown growth inhibitions against a range of Gram-positive and -negative bacteria [28]. As well, the antibacterial potential of non-polar essential oil and seed oil of coriander seeds has been established against a wide spectrum of Gram-positive and -negative bacteria. A previous study has reported that coriander essential oil is effective against *B. subtilis* (10.69 ± 0.47 mm) [7] and, simultaneously, another study has reported that seed oil is active against *E. coli*, *P. aeruginosa*, and *S. aureus* [11]. Our study similarly showed that non-polar extracts of coriander seeds are effective against the aforementioned bacteria.

#### 2.3.2. Determination of Minimum Inhibitory Concentration (MIC), Minimum Bactericidal Concentration (MBC), and MBC/MIC Ratio

The minimum inhibitory concentration (MIC) and minimum bactericidal concentration (MBC) were determined for all active coriander seed extracts (M_1_, M_2_, Et, Ace, EA, and He) identified using the well diffusion method (Table 3). The MIC is defined as the lowest concentration of antimicrobial agent that inhibits the visible growth of microorganisms after overnight incubation, thus measuring the effectiveness of bioactive extracts [29]. On the other hand, the MBC is the lowest concentration of antimicrobial agent that kills 99.9% of exposed microorganisms [29]. The MICs of all seed extracts varied from >8% to 1% (*w*/*v*) for the tested bacteria (Table 3). Considering the growth inhibition and MIC values, Ace is the most potent extract of coriander seeds in terms of antibacterial potential. The MICs obtained while treating various bacteria with the Ace extract ranged between 1 and 8% (*w*/*v*), and the obtained MBCs of this extract were equal to the MICs for all tested bacteria, showing its potential to inhibit growth and kill bacteria at the same concentration, except for *B. subtilis* and *E. coli*. The strongest effect of Ace was observed against *B. subtilis* (MIC and MBC = 1% and 2% *w*/*v*, respectively), while the weakest effect was reported against *S. typhimurium* and *P. aeruginosa* (MIC and MBC = 8% *w*/*v* for both). Additionally, the results indicate that Gram-positive bacteria are more sensitive to the Ace extract than Gram-negative bacteria tested (Table 3). A previous study reported that Gram-positive bacteria are comparatively more sensitive to essential oil of coriander seeds [30]; thus, the Ace extract may contain some essential components that may contribute to this activity. Notably, Ace has shown higher effectiveness against all tested bacteria, similar to the results obtained from the well diffusion assay (Figure 1).

Comparatively, EA exhibited less effectiveness than Ace, as its MIC values are greater than those of Ace, although it demonstrated sensitivity against all bacteria tested. This indicates that EA contains fewer antimicrobial components compared to Ace. In He, the strongest activity was recorded against Gram-positive *B. subtilis* [MIC and MBC = 4% (*w*/*v*)], whereas it demonstrated less effectiveness against all other bacteria, with MICs two times or more higher than for *B. subtilis.*

Considering the polar methanol extracts, MICs obtained after treating M_1_ and M_2_ varied between 2 and 4% (*w*/*v*) and 1 and >8% (*w*/*v*), respectively. Both extracts showed the strongest effectiveness against Gram-positive *B. subtilis* (MIC = 1% and MBC = 2% *w*/*v*), while weak or no activity was recorded for all other remaining bacteria. This study found the strongest antibacterial activity from both Ace and M_2_ against *B. subtilis* with 1% *w*/*v* MIC, indicating that Gram-positive *B. subtilis* shows significant sensitivity to coriander seeds.

The MBC/MIC ratio is important to classify the antibacterial agent as bactericidal or bacteriostatic. When the MBC/MIC ratio is less than 4–6, the antibacterial agent is regarded as bactericidal, while if the ratio is larger than 4–6, it is considered bacteriostatic [31]. If the antibacterial agent is bacteriostatic, the implication of the antibacterial agent may be limited as it cannot kill 99.9% of bacteria [32]. According to this study’s results, the MBC/MIC ratio of active extracts of coriander seeds ranged between 1 and 4 (Table 3), indicating that they are bactericidal and can potentially be included with alternative antibacterial compounds.

### 2.4. The Total Phenolic Content (TPC) and Antioxidant Capacity of Active Extracts of Coriander Seeds

**Figure 2:**
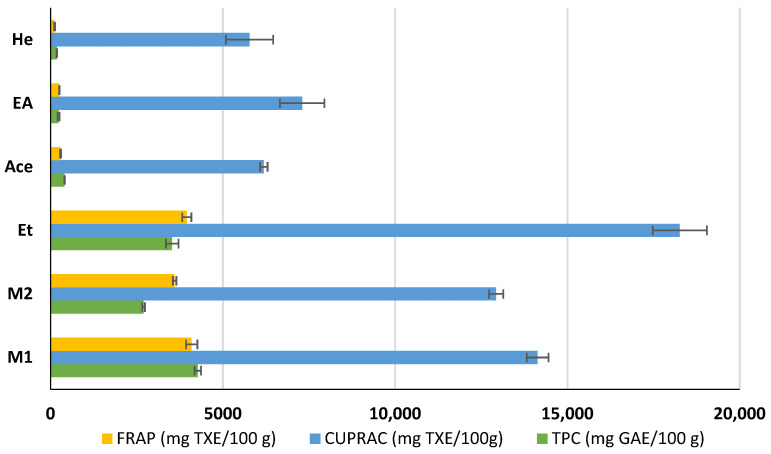
Total phenolic content (TPC) and antioxidant capacity of methanol (M_1_, M_2_), ethanol (Et), acetone (Ace), ethyl acetate (EA), and hexane (He) extracts of coriander seeds. Values are mean ± SD (n = 2). FRAP: ferric-reducing antioxidant power; CUPRAC: cupric-reducing antioxidant capacity; GAE: gallic acid equivalents; TXE: Trolox equivalents.

The TPC, CUPRAC, and FRAP of active extracts of coriander seeds were investigated. The highest TPC was found in the M_1_ extract (4273 ± 93 mg GAE/100 g DW), while the least was found in the He extract (181 ± 8 mg GAE/100 g DW). The remaining extracts exhibited the TPC values of Et > M_2_ > Ace > EA (3528 ± 183, 2698 ± 38, 404 ± 7, and 235 ± 29 mg GAE/100 g extract, respectively) (Figure 2). Our results indicated that methanol is the best solvent to extract phenolic compounds from coriander seeds, followed by ethanol. A previous study reports the TPC of methanol and hexane extracts of coriander seeds as 2921 ± 287 and 1145 ± 118 mg GAE/100 g DW, respectively [24]. This variation may be due to differences in the extraction protocols, coriander-seed variety, and geographical conditions.

The FRAP of different extracts varies as M_1_ > Et > M_2_ > Ace > EA > He (4094 ± 164, 3954 ± 133, 3599 ± 50, 290 ± 14, 254 ± 7, and 115 ± 14 mg TXE/100 g extract, respectively). The CUPRAC antioxidant capacity of the Ace, EA, and He extracts of coriander seeds shows less variation among each other at 6188, 7303, and 5774 mg TXE/100 g extract, respectively. Although the TPC of EA is lower than that of Ace, the EA shows higher CUPRAC antioxidant potential than Ace, indicating that non-phenolic antioxidants (e.g., linalool) play an important role in these extracts and may contribute to the antibacterial activity. This is because the CUPRAC test is more versatile than the FRAP test, as it can measure both the polar and non-polar compounds in plant extracts [33].

### 2.5. Identification and Quantification of Phenolic Compounds in Coriander Seed Extracts

The phenolic compounds present in different extracts were investigated using a liquid chromatography and tandem mass spectroscopy (LC–MS/MS) system with multiple reaction monitoring (MRM) targeted analysis. Thirteen phenolic compounds were identified from coriander seed extracts: gallic acid, ellagic acid, gentisic acid, caffeic acid, vanillic acid, o-coumaric acid, sinapinic acid, chlorogenic acid, salicylic acid, ferulic acid, rutin, trans-cinnamic acid, and quercetin (Table 4). Both M_2_ and Et contained the highest number of identified compounds (12 compounds), while the He extract showed the lowest number of identified compounds (5 compounds). The TPC results are reflected by the LC-MS/MS data, indicating that He has the lowest amount of coriander-seed phenolics (Figure 2). Similar to our results (Table 4), hydroxybenzoic acid (gallic, syringic, salicylic, and vanillic acids), hydroxycinnamic acid (ferulic, caffeic, chlorogenic, o-coumaric, and cinnamic acids), and flavonoids (rutin, luteolin, amentoflavone, and quercetin) have been reported in the literature for coriander seeds [17,18,34].

Quantification of phenolic compounds in coriander seed extracts is important as it reveals discriminating compounds that may have a relationship with the various biological activities. Various compounds present in seed extracts were quantified using corresponding standards and listed in Table 5. This comparative study shows different contents of phenolic compounds in coriander seed extracts that were prepared from solvents with differing polarities. The highest amount of gallic (23.32 ± 1.11 µg/g extract), ellagic (53.24 ± 1.01 µg/g extract), caffeic (856.62 ± 6.19 µg/g extract), vanillic (825.61 ± 9.53 µg/g extract), o-coumaric (526.01 ± 2.18 µg/g extract), and salicylic (30.83 ± 0.47 µg/g extract) acids, as well as quercetin (451.06 ± 17.52 µg/g extract), were reported in M_2_ compared to the other extracts. Antibacterial analysis shows that this polar extract has a good antibacterial potential (Figure 1, Table 3), and this may be attributed to a high abundance of phenolic compounds in it.

The phenolic composition of M_2_ indicates that 70% methanol and the respective extraction method are superior for extracting various categories of phenolic compounds (hydroxybenzoic acid, hydroxycinnamic acids, and flavonoids) from coriander seeds. This can be explained by the structural chemistry of major categories of phenolic compounds and methanol, and the nature of the chemical interaction between them. Methanol, which has a singular alcohol group (hydrophilic) attached to a methyl group (lipophilic), exhibits a wider range of solubility than water, although its dipole moment is marginally smaller. Hydroxybenzoic and hydroxycinnamic acids have a carboxyl group and a phenol moiety, showing strong affinity towards a polar base. Flavonoids such as quercetin have a lipophilic diphenyl propane base structure, showing affinity towards bases with less polarity. When methanol is mixed with water (e.g., 70% aqueous methanol), it allows for dissolving a wide range of polar compounds, forming intermolecular hydrogen bonds resulting in higher extraction efficiency. Additionally, the extraction method also affects the extraction efficiency of phenolics.

According to the previous study by Msaada et al. [17], the gallic acid, caffeic acid, vanillic acid, o-coumaric acid, chlorogenic acid, ferulic acid, rutin, trans-cinnamic acid, and quercetin contents of methanol extracts of coriander seeds obtained from different varieties (Tunisian, Syrian, and Egyptian) are 74.12–147.65, 6.73–87.6, 37.37–76.24, 2.61–89.87, 14.07–147.8, 43.7–108.4, 10.99–139.6, 2.43–53.35, and 2.15–52.53 µg/g extract, respectively. Comparatively, methanol extracts (M_1_ and M_2_) in our study report higher contents of caffeic acid, vanillic acid, o-coumaric acid, chlorogenic acid, ferulic acid, rutin, and quercetin, while the content of trans-cinnamic acid is in alignment with the results of Msaada et al. [17]. The variations in the individual phenolic contents could be ascribed to the differences in the origin of the seeds and extraction protocols employed. For instance, our study has used aqueous methanol, while other studies have used pure methanol instead, which may affect the extractability of phenolics to the solvent due to the difference in polarity.

The polar extract Et was reported with the highest amount of chlorogenic acid (4213.28 ± 27.33 µg/g extract), ferulic acid (457.79 ± 3.56 µg/g extract), gentisic acid (68 ± 1.66 µg/g extract), sinapinic acid (153.56 ± 3.65 µg/g extract), trans-cinnamic acid (29.77 ± 8.11 µg/g extract), and rutin (10,531.44 ± 213.47 µg/g extract), showing that 80% ethanol is a favorable solvent to extract these phenolic compounds.

Moderately polar extracts of coriander seeds, Ace and EA, have shown a majority of the identified compounds—gentisic acid, caffeic acid, vanillic acid, o-coumaric acid, sinapinic acid, chlorogenic acid, salicylic acid, ferulic acid, trans-cinnamic acid, rutin, and quercetin—albeit in lower quantities. The most abundant phenolic compound reported in Ace is vanillic acid (345.87 ± 5.71 µg/g extract), followed by quercetin (227.33 ± 21.02 µg/g extract) and caffeic acid (230.90 ± 2.7 µg/g extract). The major phenolic compounds present in EA are vanillic acid (141.13 ± 0.53), chlorogenic acid (119.59 ± 1.59 µg/g extract), and ferulic acid (114.24 ± 1.19 µg/g extract). The non-polar He has been reported with phenolic compounds such as caffeic acid, ferulic acid, o-coumaric acid, salicylic acid, vanillic acid, and rutin with trace amounts (<6 µg/g extract) (Table 5). This may be attributed to the weaker ability of these solvents to engage in intermolecular hydrogen bonding with polar phenolic compounds, since their molecular structures limit strong interactions with hydroxyl groups.

However, these extracts are more potent in terms of antibacterial activity (showing inhibition against all bacteria tested) (Figure 1), which may be due to their ability to pull out more non-polar active compounds from coriander seeds. The He extract, which showed several phenolic compounds in trace amounts, showed some antibacterial potential (Figure 1), supporting the above observation. Hexane extracts of coriander seeds have been reported with high amounts of terpenoids such as linalool, camphor, and geraniol [35]. Further research is recommended to investigate non-polar components in the Ace, EA, and He extracts.

According to this study, the most predominant hydroxybenzoic acid and hydroxycinnamic acid present in coriander seeds are vanillic acid and chlorogenic acid, respectively. The major flavonoids present in coriander seeds are rutin and quercetin. Rutin is a flavonoid glycoside, while quercetin is an aglycone flavonoid, and both compounds have exhibited many biological properties [34]. Both M_2_ (the butanol fraction of a methanol extract of coriander seeds) and Et have been reported with higher amounts of rutin and quercetin compared to other extracts (Table 5). Considering quercetin, there is no significant difference (*p* < 0.05) in quercetin content in both extracts (Table 5). In addition, the quercetin content in M_1_ and Ace is statistically similar (*p* < 0.05), indicating that both the methanol and acetone solvents are capable of extracting quercetin from coriander seeds. Conversely, rutin is reported in polar extracts in significantly higher amounts (*p* < 0.05) compared to moderate-polar and non-polar extracts, implying that polar solvents have a high potential to extract rutin from coriander seeds (Table 5).

### 2.6. Correlation Analysis

**Figure 3:**
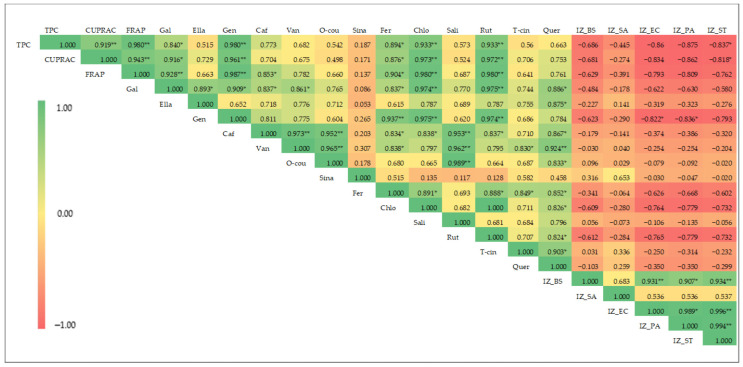
Heat map of Pearson’s correlation coefficient for phenolic content, antioxidant activity, phenolic composition, and antibacterial activity of coriander seed extracts (TPC: total phenolic content; CUPRAC: cupric-reducing antioxidant capacity; FRAP: ferric-reducing antioxidant power; Gal: gallic acid; Ella: ellagic acid; Gen: gentisic acid; Caf: caffeic acid; Van: vanillic acid; O-cou: o-coumaric acid; Sina: sinapinic acid; Fer: ferulic acid; Chlo: chlorogenic acid; Sali: salicylic acid; Rut: rutin; T-cin: trans-cinnamic acid; Quer: quercetin; IZ_BS: inhibition zone of *B. subtilis*; IZ_SA: inhibition zone diameter of *S. aureus*; IZ_EC: inhibition zone diameter of *E. coli*; IZ_PA: inhibition zone diameter of *P. aeruginosa*; IZ_ST: inhibition zone diameter of *S. typhimurium*. ** and *: significant at *p* < 0.01 and *p* < 0.05, respectively.

Pearson’s correlation coefficients (r) of the measured parameters of coriander seed extracts are shown in Figure 3. According to the results, there is a significant (*p* ≤ 0.01) and positive correlation between TPC and antioxidant activity [both CUPRAC (r = 0.92) and FRAP (r = 0.98)] of these extracts. In plant extracts, antioxidant capacity is often attributed to phenolics, which act as hydrogen donors, establishing strong positive correlations between TPC and CUPRAC, FRAP, and 2,2-diphenyl-1-picrylhydrazyl (DPPH) antioxidant activities [36,37]. The correlation analysis also showed that FRAP is significantly (*p* ≤ 0.01) and positively correlated with CUPRAC, with a correlation coefficient of 0.943.

The FRAP of the coriander seed extracts is strongly and positively correlated with the phenolic compounds—gallic acid (r = 0.928, *p* ≤ 0.01), gentisic acid (r = 0.987, *p* ≤ 0.01), caffeic acid (r = 0.853, *p* ≤ 0.05), ferulic caid (r = 0.904, *p* ≤ 0.05), chlorogenic acid (r = 0.980, *p* ≤ 0.01), and rutin (r = 0.980, *p* ≤ 0.01)—showing that a higher antioxidant potential of extract is highly correlated with higher amounts of the above phenolics. Similarly, the CUPRAC of the extracts is positively correlated with the following phenolic compounds: gallic acid (r = 0.889, *p* ≤ 0.05), gentisic acid (r = 0.961, *p* ≤ 0.01), chlorogenic acid (r = 0.973, *p* ≤ 0.01), ferulic acid (r = 0.876, *p* ≤ 0.01), and rutin (r = 0.972, *p* ≤ 0.01). The results indicate that high level of antioxidant activity of coriander seeds is correlated with high level of phenolic compounds such as gallic acid, chlorogenic acid, gentisic acid, ferulic acid, and rutin. The simultaneous use of different-polar extracts of coriander seeds provides a reliable comparison and correlation analysis among TPC, antioxidant capacity, and content of major phenolic compounds. Previous studies have reported a positive correlation between antioxidant capacity and TPC across extracts prepared from plant material using different polar solvents [38,39,40].

Negative correlations are observed between antibacterial activity and the TPC, antioxidant capacity, and phenolic composition of the extracts (Figure 3), suggesting that non-phenolic compounds may be responsible for the observed activity in coriander seed extracts. The previous literature has reported on the antibacterial potential of essential oil of coriander seeds, with linalool displaying the highest antibacterial activity [10]. Moreover, the previous literature reports that hexane solvent can extract essential-oil components from coriander seeds, such as linalool and geraniol [35], suggesting that essential-oil components may be responsible for the observed antibacterial activity in moderate-polarity and non-polar extracts of coriander seeds. Further investigations are recommended to explore the chemical composition of moderate-polarity and non-polar extracts of coriander seed.

## 3. Materials and Methods

### 3.1. Chemicals and Reagents

Folin–Ciocalteu reagent (FCR), copper (II) chloride (CuCl_2_), ammonium acetate, neocuproine (2,9-Dimethyl-1,10-phenanthroline), Trolox (6-Hydroxy-2,5,7,8-tetramethylchroman-2-carboxylic acid), ferric chloride (FeCl_3_), TPTZ (2,4,6-tris(2-pyridyl)-s-triazine), gallic acid, and gentamicin were purchased from Sigma-Aldrich, St. Louis, MO, USA. Muller Hinton Agar (MHA) was purchased from Becton Dickinson and Company, Franklin Lakes, NJ, USA. Ethanol, methanol, acetone, ethyl acetate, hexane, n-butanol, dimethyl sulfoxide (DMSO), and sodium carbonate (NaCO_3_) were of analytical grade and purchased from Chem Supply (Gillman, South Australia, Australia). All standards for the LC-MS/MS work were purchased from Sigma Aldrich (St. Louis, MO, USA) and were of analytical grade (purity > 99%; caffeic acid, chlorogenic acid, ellagic acid, gallic acid, gentisic acid, o-coumaric acid, salicylic acid, sinapinic acid, trans-cinnamic acid, vanillic acid, quercetin, and rutin). Formic acid was purchased from Sigma Aldrich (St. Louis, MO, USA). Mobile-phase solvents (LC-MS grade) were purchased from Supelco^®^ (Merck KGaA, Darmstadt, Germany).

### 3.2. Sample Collection and Preparation

Two dried-coriander-seed samples (“In” and “Hy”) were purchased from a local market in Rockhampton, Queensland, Australia. Seeds were dried overnight in an oven at 50 °C. Next, they were ground to a fine powder (<1 mm particle size) using a Breville Coffee and Spice grinder (Alexandria, New South Wales, Australia) before using it for analysis. In this study, two commercial seed samples were initially analyzed for total phenolic content and antioxidant capacity to determine which sample to use for further chemical and in vitro antibacterial assay.

### 3.3. Extraction of Phenolic Compounds and Antioxidants

Phenolic compounds and antioxidants from coriander-seed samples were extracted using 90% methanol according to a previously published method [41]. Briefly, coriander-seed powder (0.5 g) was added to 90% aqueous methanol (7 mL) (*v*/*v*), mixed upon vortexing (10 s), followed by an end-over-end mixer (RATEK, Australia) (50 rpm for 60 min). The mixture was then centrifuged (Thermo Scientific Megafuge 16, Dreieich, Germany), and the supernatant was separated. The same extraction process was repeated (20 min mixing time). The final volume of combined supernatants was made up to 15 mL with 90% methanol, and used for the total phenolic and antioxidant analyses.

### 3.4. Total Phenolic Content (TPC) of Coriander Seeds

The TPC of coriander-seed samples was determined using a modified Folin–Ciocalteu method, as previously published [41,42]. Accordingly, methanol extract of the sample (400 μL) was mixed with the Folin–Ciocalteu reagent (2 mL) diluted at 1:10 (*v*/*v*). After a 10 min incubation in darkness at room temperature, the mixture was combined with 5% *w*/*v* aqueous sodium carbonate solution (2 mL) and vortexed. The resulting samples were incubated in a water bath (at 40 °C for 10 min) and, finally, absorbance was measured at 760 nm using a UV–Visible spectrophotometer (Thermo Scientific GENESYS 10S, Burladingen, Germany). TPC of seed samples was quantified in terms of gallic acid equivalents (GAE) using a standard calibration curve ranging from 20 to 120 mg/L.

### 3.5. Antioxidant Capacity of Coriander Seeds

#### 3.5.1. Cupric-Reducing Antioxidant Capacity (CUPRAC)

The CUPRAC of coriander seeds was determined in accordance with the previous methods [43]. According to this method, to a test tube containing methanol extract (100 μL), 10 mM aqueous copper (II) chloride (1 mL), 1 M aqueous ammonium acetate (1 mL), Milli-Q^®^ water (Thermo Fisher Scientific, Massachusetts, USA) (1 mL), and freshly prepared 7.5 mM neocuproine ethanol solution (1 mL) were added, and then the mixture was vortexed for 10 s. Next, the mixture was incubated in a water bath (at 50 °C for 30 min), and the absorbance was recorded at 450 nm using a UV–Visible spectrophotometer (Thermo Scientific GENESYS 10S, Madison, WI, USA). The CUPRAC of the extract was quantified by means of Trolox (6-hydroxy-2,5,7,8-tetramethyl-chromane-2-carboxylic acid) equivalents (TRX) using a standard calibration curve ranging from 50 to 600 mg/L.

#### 3.5.2. Ferric-Reducing Antioxidant Power (FRAP)

The FRAP of the extract was determined using the method described by Johnson et al. in 2021 [44]. Initially, the FRAP reagent was freshly prepared according to the method, and then 3 mL was mixed with the extract (100 μL) and vortexed for 10 s. The mixture was incubated in a water bath (at 37 °C for 4 min) and the absorbance was measured at 593 nm using a UV–Visible spectrophotometer (Thermo Scientific GENESYS 10S, Madison, USA). The FRAP of the methanol extract was determined as a function of the equivalent absorbance of Trolox using a standard calibration curve in the range of 10–150 mg/L. Since the commercial coriander sample with the highest TPC, FRAP, and CUPRAC values was from the “In” sample, it was selected and used for further chemical and in vitro antibacterial investigations, as described below.

### 3.6. Preparation of Coriander Seed Extracts for Antibacterial Assay

Different cold extraction methods (using end-over-end shaking and magnetic stirring) were employed to prepare different extracts of coriander seeds (of the “In” sample) using different solvents (water, methanol, ethanol, acetone, ethyl acetate, and hexane) for in vitro antibacterial assays.

#### 3.6.1. Preparation of Polar Extracts (Water, Methanol, and Ethanol) of Coriander Seeds

The polar extracts of coriander seed were prepared using water, methanol, and ethanol as solvents and an end-over-end shaker as the cold extraction method to test the antibacterial activity. Water extract (W) of seeds was prepared by combining seed powder (2.5 g) with Milli-Q water (25 mL), followed by mixing with an end-over-end shaker (RATEK, Adelaide, South Australia, Australia) for 1 h. After the mixture was filtered (Whatman No. 4 filter paper, Thermo Fisher Scientific, Massachusetts, USA), the remaining residue was subjected to the same extraction procedure described above with another 25 mL of Milli-Q water. The combined filtrate was lyophilized (−54 °C, 500 m Torr) for 3 days to obtain the water extract. A similar extraction protocol was followed to prepare ethanol extract (Et) using 80% aqueous ethanol, although rotary evaporation (Buchi Rotavapor R-114, Flawil, Switzerland) was used to remove the solvent to obtain a dry ethanol extract. Two methanol extracts (M_1_ and M_2_) of coriander seeds were prepared using 90% and 70% aqueous methanol as the solvents. The M_1_ extract was prepared by combining coriander-seed powder (2.5 g) with 90% methanol (50 mL), followed by mixing using an end-over-end shaker (RATEK, Australia) for 1 h. Then the mixture was centrifuged (Thermo Scientific Megafuge 16, Dreieich, Germany), and the supernatant was decanted. The residue was re-extracted with the same extraction procedure (20 min mixing time), and the combined supernatants were filtered (0.45 µm, ADVANTEC, Bunkyo, Japan). The filtrate was rotary-evaporated (Buchi Rotavapor R-114, Switzerland) down to semisolid consistency. Finally, the residue was redissolved in Milli-Q water and lyophilized (−54 °C, 500 m Torr) for 3 days to obtain M_1_. The M_2_ extract was prepared using 70% aqueous methanol according to the previously published method described by Mechchate et al. [2], with slight modifications. Briefly, seed powder (10 g) was defatted with hexane three times (30 mL × 3), and then extracted with 70% methanol (100 mL) using an end-over-end shaker (RATEK, Australia) at 50 rpm for 60 min. The resulting mixture was filtered using vacuum filtration, and the filtrate was separated. The same extraction protocol was repeated for the remaining residue, and the combined filtrates were concentrated in vacuo, re-diluted with Milli-Q water, and then extracted with n-butanol (30 mL × 3) using a separatory funnel. The n-butanol fraction was rotary-evaporated (Buchi Rotavapor R-114, Flawil, Switzerland) to dryness to obtain the M_2_ extract. This extract (M_2_), prepared using this solvent ratio and condition, was previously reported to have anti-inflammatory and antidiabetic properties; therefore, it was used to evaluate the antibacterial properties in vitro in the present study. All polar extracts were stored at 4 °C until further use. The percentage yield (% *w*/*w*) of each extract was calculated according to Equation (1):

(1) $$Percentage\;yield\;(\%\;w/w)=\frac{weight\;of\;extract\;yielded\;(g)}{weight\;of\;coriander\;seeds\;(g)}\times 100$$

#### 3.6.2. Preparation of Semi-Polar and Non-Polar Extracts (Acetone, Ethyl Acetate, and Hexane) of Coriander Seeds

The semi-polar and non-polar extracts of seeds were prepared using hexane, ethyl acetate, and acetone as solvents and magnetic stirring as the extraction technique. Seed powder (25.00 g) was placed in a 500 mL conical Erlenmeyer flask, and 250 mL of hexane was added. Next, the mixture was stirred using a magnetic stirrer (IEC, Thornbury, Victoria, Australia) at room temperature (25 °C) for 48 h. Subsequently, the hexane extract was filtered (Whatman No. 4 filter paper) under vacuum, and then the hexane was removed using a rotary evaporator (Buchi Rotavapor R-114, Switzerland) to obtain the hexane extract (He). A similar method was followed to prepare ethyl acetate (EA) and acetone (Ace) extracts from seeds. The percentage yield of each extract was calculated (Equation (1)) and stored at 4 °C until further use.

### 3.7. In Vitro Antibacterial Activity of Coriander Seeds

#### 3.7.1. Preparation of Agar Plates

The culture media was Muller Hinton Agar (MHA) (BD™ Difco™), and this was dissolved (38 g) in RO water (1 L) and then autoclaved (121 °C) for 20 min. After cooling, the sterilized agar solution was put in a water bath at 54 °C for 10 min before pouring into sterilized Petri dishes (25 mL for each). The MHA plates were dried appropriately before using them for the experiments.

#### 3.7.2. Culture and Maintenance of Test Bacteria

The antibacterial activity of coriander seeds was carried out on Gram-positive and -negative food-borne pathogenic bacteria. *Staphylococcus aureus* and *Bacillus subtilis* were used as Gram-positive bacteria while *Escherichia coli*, *Salmonella typhimurium*, and *Pseudomonas aeruginosa* were used as Gram-negative bacteria for the assay. They were kindly provided by the School of Health, Medical and Applied Sciences, CQUniversity, Rockhampton, Australia.

#### 3.7.3. Sample Preparation for Antibacterial Activity

All extracts prepared in Section 3.6 were used to make a concentration of 30% (*w*/*v*) to screen for antibacterial activity. Antibacterial activity was investigated using the extracts prepared under Section 3.6 at a concentration of 30% (*w*/*v*). The W, M_1_, M_2_, and Et extracts were dissolved in 10% DMSO, while Ace, EA, and He were dissolved in DMSO, considering their solubility. Each extract (300 mg) was dissolved in its respective solvent (1 mL) in sterile 1.5 mL Eppendorf tubes using sonication (20 min) in a sonicated water bath (Soniclean Ultrasonic cleaner, Dudley Park, South Australia, Australia) and freshly used for the antibacterial assay.

#### 3.7.4. Agar-Well Diffusion Method

The antibacterial activity of coriander seed extracts was investigated using the agar-well diffusion method in Mueller Hinton Agar (MHA) plates, according to the method described by Manandhar et al. [45]. Firstly, bacterial inoculum was prepared by suspending pure bacterial colonies in the sterilized saline water (0.9% NaCl) until the turbidity matched a 0.5 McFarland standard (1.5 × 10^8^ CFU/mL). The prepared bacterial inocula were used to inoculate the agar plates with the lawn culture technique. Using a sterile cork-borer, 6 mm wells were bored in the inoculated agar plates, and each seed extract (35 µL) filled into the wells. The agar plates filled with extracts were left at room temperature for 30 min and then incubated for 24 h at 37 °C. After incubation, the inhibition zone diameter (IZD) corresponding to each well was recorded using a ruler. Gentamicin was used as a positive control while DMSO was used as the negative control for all bacteria tested. The experiment was carried out in triplicate, and the mean IZD was obtained.

#### 3.7.5. Minimum Inhibitory Concentration (MIC)

Seed extracts active in the agar-well diffusion assay (M_1_, M_2_, Et, Ace, EA, and He) were selected and used for the evaluation of the MIC. Initially, stock solutions were prepared from each seed extract using 10% DMSO and the sonication method (20 min). Stock solutions were prepared at a concentration of 32% (*w*/*v*) for MIC evaluation, as all active extracts exhibited an inhibition zone at this concentration.

The minimum inhibitory concentrations of seed extracts were investigated using the broth microdilution method in 96-well plates (SARSTEDT, Nümbrecht, Germany) following the method described by Van et al. [46]. Two-fold serial dilutions were made in 96-well plates using the stock solution in Muller Hinton broth (MHB) (BD™ Difco™), with dilutions ranging from 16% to 0.25% (*w*/*v*). The final concentration of DMSO in all wells was less than 2.5% in order to avoid any solvent impact upon the bacteria [47]. Bacterial inocula were prepared by the direct colony suspension method by suspending isolated bacterial colonies (from 24 h bacterial cultures) in sterile saline solution (0.9% NaCl) to give the turbidity equivalent to that of 0.5 McFarland standard [1 × 10^8^ colony-forming units (CFU)/mL]. Then, these inocula were diluted 1:100 using sterile MHB to obtain test bacterial suspensions (0.5 × 10^6^ CFU/mL), and used for the experiment within 15 min after preparation. The test bacterial suspension (50 µL) was inoculated to each well containing 50 µL of diluted plant extract. The 96-well plates were incubated at 37 °C for 24 h. The lowest concentration that does not show visible growth of bacteria was determined as the MIC for each extract. Bacterial growth was visually determined as any turbidity in wells; hence, the lowest concentration without turbidity was recorded as the MIC value of each extract.

#### 3.7.6. Minimum Bactericidal Concentration (MBC)

The MBC of extracts active for the antibacterial assay was investigated using the method described by Mogana et al. [48]. First, the MIC experiments for extracts were carried out as described in Section 2.3.2. Then, 10 µL aliquots from the well corresponding to the MIC value, as well as two wells above the MIC-value well, were spread on MHA agar plates using a spreader and, subsequently, incubated at 37 °C for 24 h. After the incubation, the number of colonies in each agar plate was enumerated. The MBC value of each extract was recorded as the lowest concentration of extract that prevents the growth of the bacteria after subculturing on agar plates [29]. Each experiment was carried out in duplicate.

### 3.8. Total Phenolic Content and Antioxidant Capacity of Active Seed Extracts

Active extracts of coriander seeds (M_1_, M_2_, Et, Ace, EA, and He) were subjected to TPC, CUPRAC, and FRAP analyses following the procedures described in Section 3.4 and Section 3.5, respectively. Each extract (20 mg) was dissolved in 90% methanol (4 mL) and this was used for the total phenolic and antioxidant assays.

### 3.9. LC-MS/MS Analysis of Phenolic Compounds

Targeted quantification of phenolic acids and flavonoids present in coriander seed extracts (prepared using the “In” sample) that returned positive results for the antibacterial assays was performed on a Waters ACQUITY™ I-Class UPLC (Binary Solvent Manager + Sample Manager FTN) coupled to a Xevo™ TQ-S micro-triple–quadrupole mass spectrometer (Waters Corporation, Milford, MA, USA). Separation of phenolic compounds was performed on an ACQUITY™ Premier BEH C18 column (2.1 × 150 mm, 1.7 µm; Waters Corporation, Milford, MA, USA). Chromatographic separation was achieved using two complementary gradient programs. Method A (for phenolic acids) used Milli-Q water (0.1% Formic acid) (A) and methanol (0.1% Formic acid) (B) at 0.20 mL min^−1^ with a gradient from 20% B to 98% B over 10 min, held for 3 min, then returned to 20% B and re-equilibrated for a total run time of 16 min. The column was held at 30 °C. Method B (for flavonoids) used Milli-Q water (0.1% Formic acid) (A) and Acetonitrile (0.1% Formic acid) (B) at 0.20 mL min^−1^ with a gradient from 6% B to 98% B over 21 min, held for 2 min, and re-equilibrated at 6% B for a total run time of 25 min. The column temperature was 45 °C. For both methods, injection volumes were 1.0 µL for the samples/blanks and 0.2–1.0 µL for the standards to enable calibration curve dilution. The Xevo TQ-S micro was operated in Multiple Reaction Monitoring (MRM) mode with electrospray ionization. For Method A (ESI^−^), the capillary voltage was 1.0 kV, source temperature 150 °C, desolvation temperature 500 °C, cone gas 50 L h^−1^, and desolvation gas 1000 L h^−1^ (N_2_). For Method B (ESI^+^), the capillary voltage was 1.0 kV, source temperature 120 °C, desolvation temperature 350 °C, cone gas 50 L h^−1^, and desolvation gas 800 L h^−1^ (N_2_). Argon served as the collision gas. Detector gains were 1.0 (ESI^−^) and 0.5 (ESI^+^). Dwell times were automatically optimized (~11 ms in ESI^−^; ~2 ms in ESI^+^). External mixed-standard calibration curves (0.25–20.0 mg L^−1^; r^2^ > 0.99) were used for quantification, and the final results expressed as µg g^−1^ extract. MRM transitions for the target analytes are displayed in Table S1 (Supplementary Materials).

## 4. Statistical Analysis

All data except the LC-MS/MS data were analyzed by descriptive statistics using SPSS software version 29. The data were expressed as mean ± SD of duplicate chemical assays and triplicate antibacterial testing. Pearson’s correlation analysis was for data obtained from the chemical assays and bioactivity assays, and was carried out at a 95% confidence level. The *p* values ≤ 0.05 were considered significant. Univariate analysis of LC-MS/MS data was performed using GraphPad Prism version 9.4.1 for Windows (GraphPad Software, San Diego, CA, USA). One-way ANOVA and Tukey’s post hoc analysis was conducted using a confidence interval of *p* < 0.05.

## 5. Conclusions

The polar extracts of coriander (*Coriandrum sativum* L.) seeds are rich in phenolic compounds and antioxidants, while the moderate-polarity and non-polar extracts of coriander seeds possess antibacterial potential that may be attributed to non-polar compounds. The TPC of the coriander seed extracts is positively and significantly correlated with antioxidant activity, revealing phenolic compounds as the main contributors to antioxidant potential. Furthermore, antioxidant activity is positively correlated with chlorogenic acid, trans-ferulic acid, and rutin, showing that they are the major contributors to the antioxidant activity of coriander seeds. The antibacterial assay demonstrated that the acetone extract has the highest antibacterial potential compared to other extracts, and Gram-positive *B. subtilis* is the bacterium most sensitive to coriander seed extracts, regardless of the extraction solvent. The negative correlation between the antibacterial activity and phenolic/antioxidant data may be indicative of the antibacterial role of non-polar compounds in the coriander seeds. Finally, further research is recommended to investigate the non-polar antibacterial compounds of coriander seeds.

## Figures and Tables

**Table 1:** TPC and antioxidant capacity of coriander seeds.

Commercial Coriander Sample	TPC mg GAE/100 g DW	FRAP mg TRX/100 g DW	CUPRAC mg TRX/100 g DW
In	274 ± 3	424 ± 13	1142 ± 13
Hy	161 ± 3	279 ± 10	620 ± 20

Data represent means of duplicate analysis ± SD; CUPRAC: cupric-reducing antioxidant capacity; FRAP: ferric-reducing antioxidant power.

**Table 2:** Extraction yield of coriander seed extracts prepared from different solvents.

Coriander Seed Extract	Extraction Yield % (*w*/*w*) DW
W	9.3
M_1_	7.1
M_2_	2.2
Et	12.1
Ace	7.2
EA	9.5
He	12.0

M_1_ and M_2_; methanol extracts, Et; ethanol extract, Ace; acetone extract, EA; ethyl acetate extract, He; hexane extract.

**Table 3:** The minimum inhibitory concentrations (MIC), minimum bactericidal concentrations (MBC), and MBC/MIC ratios of coriander seed extracts.

Test Bacteria	Minimum Inhibitory Concentration (MIC)						
	M_1_ (% *w*/*v*)	M_2_ (% *w*/*v*)	Et (% *w*/*v*)	Ace (% *w*/*v*)	EA (% *w*/*v*)	He (% *w*/*v*)	Gen (% *w*/*v*)
BS	2	1	4	1	2	4	1.25 × 10^−5^
SA	4	2	4	2	2	8	2.5 × 10^−5^
EC	-	8	-	4	8	8	10 × 10^−5^
PA	-	>8	-	8	>8	>8	5 × 10^−5^
ST	-	8	-	8	>8	-	2.5 × 10^−5^
	**Minimum Bactericidal Concentration (MBC)**						
	**M_1_** **(% *w*/*v*)**	**M_2_** **(% *w*/*v*)**	**Et** **(% *w*/*v*)**	**Ace** **(% *w*/*v*)**	**EA** **(% *w*/*v*)**	**He** **(% *w*/*v*)**	
BS	4	2	4	2	2	4	1.25 × 10^−5^
SA	8	8	8	2	4	8	5 × 10^−5^
EC	-	8	-	8	8	8	10 × 10^−5^
PA	-	>8	-	8	>8	>8	20 × 10^−5^
ST	-	>8	-	8	>8	-	5 × 10^−5^
	**MBC/MIC ratio**						
	**M_1_**	**M_2_**	**Et**	**Ace**	**EA**	**He**	
BS	2	2	1	2	1	1	1
SA	2	4	2	1	2	1	2
EC	-	1	-	2	nd	1	1
PA	-	nd	-	1	nd	nd	4
ST	-	nd	-	1	nd	nd	2

BS: *Bacillus subtilis*; SA: *Staphylococcus aureus*; EC: *Escherichia coli*; PA: *Pseudomonas aeruginosa*; ST: *Salmonella typhimurium*; M_1_ and M_2_: methanol extracts; Et: ethanol extract; Ace: acetone extract; EA: ethyl acetate extract; He: hexane extract; Gen: gentamicin; nd: not determined.

**Table 4:** Chemical structures of phenolic compounds identified in extracts of coriander seeds.

Number	Phenolic Compound	Compound Category	Retention Time
1	Gallic acid 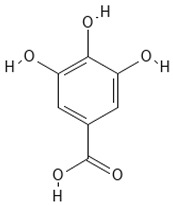	Hydroxybenzoic acid	2.48 min
2	Ellagic acid 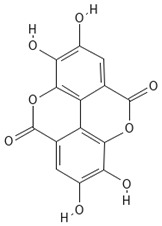	Hydroxybenzoic acid	4.62 min
3	Gentisic acid 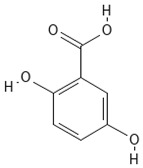	Hydroxybenzoic acid	5.18 min
4	Caffeic acid 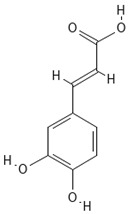	Hydroxycinnamic acid	5.5 min
5	Vanillic acid 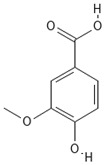	Hydroxybenzoic acid	5.51 min
6	O-coumaric acid 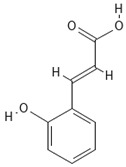	Hydroxycinnamic acid	6.26 min
7	Sinapinic acid 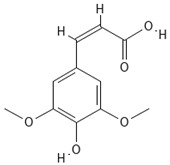	Hydroxycinnamic acid	6.28 min
8	Ferulic acid 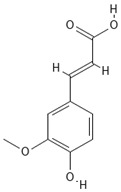	Hydroxycinnamic acid	6.41 min
9	Chlorogenic acid 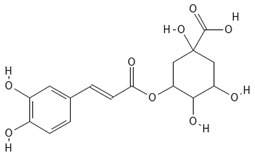	Hydroxycinnamic acid	7.00 min
10	Salicylic acid 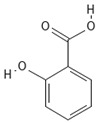	Hydroxybenzoic acid	8.23 min
11	Rutin 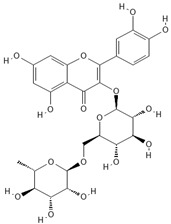	Flavonoid	8.28 min
12	Trans-cinnamic acid 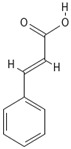	Cinnamic acid	9.24 min
13	Quercetin 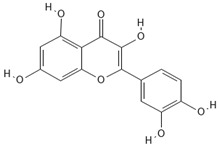	Flavonoid	10.73 min

**Table 5:** Quantification of different phenolic compounds in coriander seed extracts active in antibacterial assay.

Compound	M_1_	M_2_	Et	EA	Ace	He
Gallic acid	13.32 ± 1.07 ^b^	23.32 ± 1.11 ^a^	22.99 ± 2.39 ^a^	n.d.	n.d.	n.d.
Ellagic acid	n.d.	53.24 ± 1.01 ^a^	52.06 ± 1.37 ^a^	n.d.	n.d.	n.d.
Gentisic acid	63.32 ± 4.39 ^a^	49.87 ± 0.30 ^b^	68.00 ± 1.66 ^a^	n.d.	10.44 ± 0.16 ^c^	n.d.
Caffeic acid	523.02 ± 7.11 ^b^	856.62 ± 6.19 ^a^	441.72 ± 2.70 ^c^	38.71 ± 4.96 ^e^	230.90 ± 2.70 ^d^	6.15 ± 0.31 ^f^
Vanillic acid	444.11 ± 6.13 ^c^	825.61 ± 9.53 ^a^	505.44 ± 8.60 ^b^	141.13 ± 0.53 ^e^	345.87 ± 5.71 ^d^	4.51 ± 0.62 ^f^
o-Coumaric acid	211.03 ± 2.45 ^b^	526.01 ± 2.18 ^a^	191.87 ± 1.86 ^c^	44.12 ± 0.05 ^e^	171.38 ± 2.44 ^d^	1.26 ± 0.18 ^f^
Ferulic acid	429.54 ± 1.51 ^b^	384.90 ± 9.63 ^c^	457.79 ± 3.56 ^a^	114.24 ± 1.19 ^e^	259.95 ± 2.90 ^d^	3.34 ± 0.14 ^f^
Sinapinic acid	113.30 ± 3.25 ^b^	62.69 ± 0.50 ^c^	153.56 ± 3.65 ^a^	16.43 ± 0.68 ^e^	37.98 ± 0.79 ^d^	n.d.
Chlorogenic acid	3131.93 ± 40.03 ^c^	3468.31 ± 42.32 ^b^	4213.28 ± 27.33 ^a^	119.59 ± 1.59 ^d^	73.80 ± 1.13 ^e^	n.d.
Salicylic acid	15.36 ± 0.79 ^b^	30.83 ± 0.47 ^a^	12.76 ± 0.13 ^c^	7.38 ± 0.11 ^e^	10.50 ± 0.09 ^d^	1.59 ± 0.19 ^f^
Rutin	7827.25 ± 170.51 ^c^	8697.93 ± 31.87 ^b^	10,531.44 ± 213.47 ^a^	257.08 ± 0.79 ^d^	77.91 ± 4.79 ^e^	5.23 ± 0.66 ^f^
Trans-cinnamic acid	15.43 ± 0.68 ^b^	25.95 ± 4.29 ^a^	29.77 ± 8.11 ^a^	12.76 ± 4.66 ^b^	20.88 ± 4.63 ^a^	n.d.
Quercetin	194.43 ± 20.30 ^b^	451.06 ± 17.52 ^a^	423.82 ± 49.00 ^a^	n.d.	227.33 ± 21.02 ^b^	n.d.

Values are mean ± SD (n = 3). Means in rows followed by the same letter(s) are not significantly different at *p* < 0.05; n.d.: not detected.

## Data Availability

The original contributions presented in this study are included in the article/supplementary material. Further inquiries can be directed to the corresponding author.

## Supplementary Materials

The following supporting information can be downloaded at https://www.mdpi.com/article/10.3390/molecules30224387/s1, Table S1; MRM transitions for LC-MS/MS quantification of phenolic compounds in coriander seed extracts.
