# Supplementary material for: Targeted Metabolomic Analysis Reveals Solvent-Dependent Phenolic Variation and Associated Antioxidant and Antibacterial Activity in Coriander Seeds (Coriandrum sativum L.)
Source: Molecules. 2025 Nov 13;30(22):4387. doi: 10.3390/molecules30224387 (PMC12655154; doi:10.3390/molecules30224387)
Supplement: Supplementary file 1 [file molecules-30-04387-s001.zip › molecules-3864675-supplementary.pdf]

Supplementary material 01 (Table S1); MRM transitions for LC-MS/MS quantification of phenolic compounds in coriander seed extracts

**Analyte**	**Polarity**	**Precursor (m/z)**	**Product (m/z)**	**Cone (V)**	**Collision (V)**	**Notes**
Caffeic acid	–	178.85	134.91	23	15	(Method A)
Chlorogenic acid	–	352.98	190.97	23	15	(Method A)
Ellagic acid	–	300.83	144.91	80	34	(Method A)
Gallic acid	–	168.84	124.85	23	13	(Method A)
Gentisic acid	–	152.86	108.91	18	12	(Method A)
o-Coumaric acid	–	162.86	118.90	18	13	(Method A)
Salicylic acid	–	136.87	92.93	18	15	(Method A)
Sinapinic acid	–	222.92	207.90	23	13	(Method A)
trans-Cinnamic acid	–	146.81	102.92	40	6	(Method A)
Vanillic acid	–	166.98	151.93	23	12	(Method A)
Quercetin	+	303.10	153.00	55	30	(Method B)
Rutin (Quercetin-3-O-rutinoside)	+	611.21	303.00	23	20	(Method B)
